# Expression Pattern of Genes in Condyloma Acuminata Treated with *Clinacanthus nutans* Lindau Cream versus Podophyllin

**DOI:** 10.1155/2021/5579520

**Published:** 2021-09-17

**Authors:** Jiraporn Jantaravinid, Sukhum Jiamton, Chatchawan Srisawat, Bhoom Suktitipat, Napatara Tirawanchai

**Affiliations:** ^1^Department of Biochemistry, Faculty of Medicine Siriraj Hospital, Mahidol University, Bangkok 10700, Thailand; ^2^Department of Dermatology, Faculty of Medicine Siriraj Hospital, Mahidol University, Bangkok 10700, Thailand; ^3^Integrative Computational BioScience Center, Mahidol University, Nakhon Prathom 73170, Thailand

## Abstract

*Clinacanthus nutans* Lindua (*C. nutans*), a strong antiviral traditional medicine, can be used to treat condyloma acuminata (CA) caused by the human papillomavirus (HPV). However, its molecular mechanism for CA elimination is unknown. Herein, we conducted a randomized clinical trial to evaluate the effectiveness of *C. nutans* and its molecular mechanism compared with podophyllin, the gold standard treatment. Using a randomized block design, six patients were treated with *C. nutans* and podophyllin for four weeks. Efficacy of drugs was assessed by size reduction of the warts and HPV viral load quantification using droplet digital PCR. The gene expression profiling of CA was analyzed using NanoString Technology. After the podophyllin and *C. nutans* treatments, CA lesion sizes were reduced to 97.0% and 84.4% clearance, and the HPV viral loads were reduced by 74.0% and 46.6%, respectively. The gene expression pattern of immune profiling showed that 23 genes (i.e., *HLA-DPB, CCL3, CXCL2, CXCR2*, and *OSM*) were significantly differentially expressed by podophyllin, whereas 2 genes (*IFNL1* and *IRF2*) were remarkably expressed by *C. nutans.* In inflammatory profiling, 108 genes (i.e., *CXCL2*, *IL8*, and *STAT3*) were highly expressed by podophyllin, but none of genes were observed to change expression by *C. nutans.* These results suggested that podophyllin may reduce the HPV infection through a mechanism related to proinflammatory response. In addition, *C. nutans* was found to suppress the HPV infection through mechanism related to the activation of immune response. This study shows novel therapeutic mechanisms of podophyllin and *C. nutans*. It is suggested that *C. nutans* might be used as an alternative treatment for CA treatment.

## 1. Introduction

Human papillomavirus (HPV) is etiologically associated with the development of genital warts. More than 100 different types of HPV have been classified as either low risk (LR-HPV) or high risk (HR-HPV), based on their oncogenic characteristics [1]. Condyloma acuminata (CA), or genital warts, are mainly attributable to the LR-HPV type 6 (89%) and HPV type 11 (11%) [2]. Up to date, there are no antiviral drugs for HPV infection. Thus, therapeutic managements were not targeted at antiviral therapies but either attempt to remove physical lesion or stimulate immune response to eliminate viral infection [3]. Alternatively, medicinal plants have shown their potential to treat CA, such as podophyllin and polyphenon E (sinecatechins 15% ointment).

*Clinacanthus nutans* Lindau (*C. nutans)* is an important traditional medicine from Acanthaceae family that has been used as a vital medicine in tropical Asia. *C. nutans* has been proved to have anti-inflammatory, antioxidant, immune response and antiviral activities [4, 5]. Furthermore, it has been reported that *C. nutans* compound inhibits HPV infections by interfering at the early step of an HPV16 infection, and it may have a TLR4-transcriptional inducible effect, which was a key regulatory molecule to stimulate the immune response and inflammatory cytokines secretion [6]. Additionally, it is revealed that *C. nutans* could affect alteration in the nonspecific cell-mediate immune response, which relates to the releasing of *IL4* from peripheral blood mononuclear cell [5]. In Thailand, *C. nutans* cream has been included in the National Lists of Essential Medicines for skin inflammations treatment from virus infection [7].

To date, efficacy of *C. nutans* cream for CA treatment has not yet been studied in clinical trials nor has molecular mechanisms been addressed. Hence, this study aims to investigate the efficacy of *C. nutans* treatment on HPV-infected CA patients in comparison with podophyllin. Size reduction of the warts together with the amount of HPV DNA and gene expression profiling in response to drug treatments were subjected in the study.

## 2. Materials and Methods

### 2.1. Patients

Six Thai male patients (aged >18 years) with clinical CA diagnosis at their first visit were enrolled into this study. Each patient must have at least two CA, with at least one centimeter apart between each wart. Patients had no medical history of immunomodulator or systematic antiviral drugs usage. Patients with documented HIV or autoimmune diseases were excluded. Written informed consent according to the Declaration of Helsinki approved by The Institution Review Board (IRB), Faculty of Medicine Siriraj Hospital, Mahidol University (COA no: Si 629/2016) were obtained from all patients and were registered in Thai Clinical Trials Registry (TRTC: 062 875 7627).

### 2.2. Specimen and Sample Preparation

CA specimens were obtained from Dermatology Outpatients Unit at Siriraj Hospital, Mahidol University. Sample swabs were collected from both pretreatment and posttreatment visits. Samples were arranged in randomized block design. Each block was arranged in the block of four. Following random separation of the warts as “A” or “B,” they were treated with *C. nutans* cream (The Government Pharmaceutical Organization, Thailand, GPO) or podophyllin (Vidhyasom, Thailand). Patients were followed up every week for one month, and sample swabs were collected at week 0 and week 4. All sample swabs were preserved in DNA/RNA shield™ collection tubes at −20°C. Following extraction, DNA and RNA samples were subjected to HPV genotyping, HPV viral load, and gene expression profiling analysis.

### 2.3. Nucleic Acid Extraction

Genomic DNA and total RNA were extracted from the swab samples using the Allprep DNA/RNA Mini Kit (QIAGEN Ltd., UK). Concentration of the purified DNA was measured by a Nanodrop™ 8000 spectrophotometer and stored at −20°C until use. DNA samples with 260/280 nm absorbance ratio between 1.8 and 2.0 were diluted to approximately 5 ng/*μ*L prior to ddPCR. RNA concentration was also measured, and its purity was approximately 2 by NanoPhotometer® before gene expression analysis was immediately performed.

### 2.4. Linear Array HPV Genotyping Assay

Linear array HPV genotyping assay was performed by the Molecular Microbiology Laboratory, Department of Microbiology, Faculty of Medicine Siriraj Hospital, Mahidol University using the linear array HPV genotyping test (Roche Diagnostics, Switzerland).

### 2.5. Droplet Digital PCR (ddPCR)

HPV L1 gene was determined using QX200 ddPCR system (Bio-Rad) in comparison with human *β*-globin gene as a reference gene. Reactions were set up according to the manufacturer's instructions. All primers and ddPCR conditions were shown in Table S1. Using QX200 Droplet reader™, ddPCR droplets were analyzed by QuantaSoft™ analysis software (version 1.7.4.0917). Quantitative measurement represented the copy number/*μ*L of the PCR reaction for the HPV target gene in comparison with *β*-globin gene.

### 2.6. NanoString nCounter Gene Expression Assay and Data Analysis

Immune and inflammatory panels were selected for gene expression analysis using NanoString nCounter® Platform (NanoString Technology). The reactions were set up according to the nCounter XT Gene expression protocol. Gene expression analysis was performed using nSolver Analysis Software v4.0 (NanoString Technology) involving in 770 genes using immune panel (*NS_CANCERIMMUNE_V1.1)* and a custom designed set of 256 genes in inflammatory panel (*NS_INFL_HS_V2_C2534)*. A background correction was made by subtracting the background thresholds of negative control from raw counts. Adjusted raw counts were normalized using the combination of positive controls and reference gene normalization. The positive control normalization was computed by the geometric mean normalization factor, whereas reference gene normalization was calculated by the geometric mean of 11 genes, *SF3A3, MRP55, TLK2, HDAC3, PPIA, TBP, ZNF143, DNAJC14, ABCF1 SAP130*, and *EDC3*, in immune panel and four genes, *CLTC, CUSB, HPRT1*, and *PGK1*, in inflammatory panel.

### 2.7. Statistical Analysis

SPSS Statistic version 18 Software (IBM, USA) was used for clinical data and the viral load measurement. Pre- and posttreatments using podophyllin or *C. nutans* were compared by nonparametric Wilcoxon signed-rank test. Results were considered significant at *p* value < 0.05. Data on gene expression assay were normalized using nSolver 4.0 according to the manufacturer recommendations. Two-tailed Student's *t* tests were used to evaluate significant differences in gene expression between pre- and posttreatments using podophyllin and *C. nutans*. The *p-*values were adjusted using false discovery rate (FDR) method for multiple comparison (Benjamini–Hochberg) [8, 9]. FDRs less than 0.05 were considered significantly different. Volcano plot was created to depict differential gene expression between pre- and posttreatments on their original scale. Data were reported as a fold change in gene expression.

## 3. Results

### 3.1. HPV Genotyping from CA Lesions

Prevalence of HPV genotypes from six patients revealed six different genotypes, which were classified into LR-HPV and HR-HPV. The most common HPV genotype was LR-HPV. Only one out of six patients had both LR-HPV and HR-HPV genotypes. Regarding to the pattern of the HPV infections, four patients (66.66%) had single HPV genotype, whereas two patients (33.33%) revealed multiple HPV genotypes. Of four patients with single HPV genotype, HPV-6 was found in three patients (75%), whereas HPV-11 was found in only one patient (25%). In contrast, two out of the six patients had multiple HPV genotypes. One patient had two HPV genotypes (HPV-11 and HPV-40), and another had four HPV genotypes (HPV-11, HPV-81, HPV-45, and HPV-58). In case of multiple infections, HPV-11 was dominantly detected, with minor infection from other HPV genotypes. Thus, it is demonstrated that HPV-6 and HPV-11 were dominantly detected (Table 1).

### 3.2. Clinical Outcomes and HPV Viral Load Clearance

Baseline clinical outcome and HPV viral load clearances were described in Table 1. Five patients received podophyllin and *C. nutans* treatment were classified into 2 groups; responder (wart size and viral load were approximately >75% reduction) and nonresponder (wart size and viral load were <75% reduction). Based on the CA clearance, median of the total clearances for responder of podophyllin and *C. nutans* treatment groups were 97% and 84.85% size reduction area respectively (*p* < 0.05, Wilcoxon signed-rank test, *n* = 5). However, one patient (ID 03), was classified as nonresponder showed that lesion were not completely cured.

It is shown from the HPV viral load quantification that medians of HPV viral load of the pre- and postpodophyllin treatments were 1.74 and 0.34 HPV copies/cell, respectively, in the responder group (*p*=0.043, Wilcoxon signed-rank test; *n* = 5). As to responder of *C. nutans* treatment, median values of HPV viral load were reduced from 1.74 to 0.71 HPV copies/cell, and difference was statistically significant (*p*=0.043, Wilcoxon signed rank test, *n* = 5). For nonresponder group, which has viral load reduction less than 75%, only one patient (ID03) showed a slight decrease in HPV viral load after podophyllin treatment, but the HPV viral load greatly increased after *C. nutans* treatment. The rates of HPV viral load clearances after podophyllin and *C. nutans* treatments were 74% and 46.58%, respectively. These data were found to be significantly different (*p*=0.043; Wilcoxon signed-rank test, *n* = 5) (Table 1, Files S1).

### 3.3. Immune Gene Expression Profiling of CA after Podophyllin or *C. nutans* Treatments

**Figure 1:**
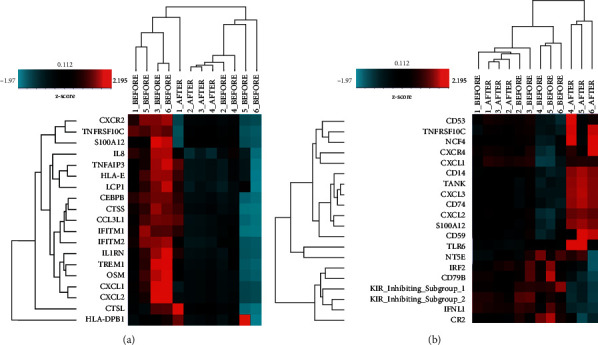
Immune gene profiling of pre- and post-treatment group as measured by NanoString Technology. (A) Heatmap of significantly altered gene expression levels by Z‐score of podophyllin treatment. (B) Heatmap of significantly altered gene expression levels by Z‐score of *C. nutans* treatment. The number horizontal bar along top is annotated by the patient samples. The red color indicates an increases in the expression of genes, and the blue color indicates a decrease in gene expression.

It was observed from the hierarchical cluster analysis using expression ratio that patterns of the gene expression between pre- and posttreatment of podophyllin and *C. nutans* were significantly different (Figure 1). In the podophyllin treatment group, there were 23 significantly differentially expressed genes identified by volcano plot filtering (adjusted *p* < 0.05; fold change >1.2) (Figure S1). Among them, two genes were upregulated and 21 genes were downregulated (Table S2). It is suggested that efficacy of podophyllin treatment might affect expression of the immune genes, for example cytokine, cell function, regulation, antigen processing, pathogen defense, TNF Superfamily, and cytotoxic gene sets. For *C. nutans* treatment group, there were only two differentially expressed genes identified by the volcano plot filtering (adjusted *p* < 0.05; fold change>2) (Figure S2). These two genes were upregulated, and no gene was downregulated (Table S3). Changing in the state from before *C. nutans* treatment to after *C. nutans* treatment was observed as less-separated cluster by differences in expression profiles of genes. These results demonstrated that efficacy of *C. nutans* treatment might affect only on some genes relating to cytokine and chemokine.

Furthermore, the differentially expressed genes in the podophyllin treatment group were compared with those in the *C. nutans* treatment group using Venn diagram (File S2). At posttreatment, 23 genes were differentially expressed for podophyllin, and only 3 genes for *C. nutans.* Interestingly, only *IFNL1* gene was upregulated in both podophyllin and *C. nutans* treatments. Additionally, *HLA-DPB1* gene was shown to be significantly upregulated in podophyllin treatment, whereas *IFNL1, IRF2* genes were found to be significantly upregulated in *C. nutans* treatment. On the contrary, 21 genes were found to be downregulated in podophyllin treatment only.

### 3.4. Inflammatory Gene Expression Profiling of CA after Podophyllin or *C. nutans* Treatments

**Figure 2:**
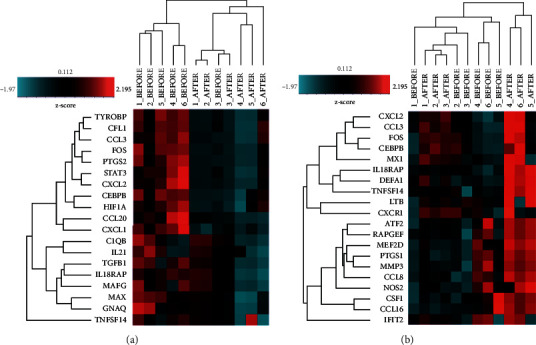
Inflammatory gene profiling of pre- and post-treatment group as measured by NanoString technology. (a) Heatmap of significantly altered gene expression levels by Z‐score in of podophyllin treatment. (b) Heatmap of altered gene expression levels by Z‐score of *C. nutans* treatment. The number horizontal bar along top is annotated by the patient samples. The red color indicates an increases in the expression of genes, and the blue color indicates a decrease in gene expression.

It was demonstrated from the heatmap hierarchical clustering that there was a differential expression of genes in the inflammatory pathway between pre- and posttreatment of both drugs (Figure 2).

Surprisingly, it was identified from the volcano plot filtering that 108 genes were significantly upregulated in podophyllin-treated CA (adjusted *p* < 0.05; fold change>1.20) (Figure S3, Table S4). This implied that podophyllin might affect the expression of genes involved in inflammatory pathway. In *C. nutans*-treated group, 58 genes were shown to be differentially expressed in a volcano plot filtering (adjusted *p* ≤ 0.10; fold change>1.20). However, none of the inflammatory genes were found to be differentially expressed if *p* < 0.05 was adjusted (Figure S4, Table S5). It is observed that the expression profiles of those genes were not significantly different between pre- and post-*C. nutans* treatments. Hence, it is suggested that *C. nutans* might not affect the inflammatory response pathway.

## 4. Discussion

HPV is etiologically associated with the development of virtually all CA. It is widely accepted that HPV-6 and HPV-11 are dominant LR-HPV in the pathogenesis of CA [10]. Our results showed that the most common types of HPV identified in CA lesions were HPV-6 and HPV-11, which was consistent with the previous studies [11, 12]. However, multiple infection was also detected in some CA lesions (ID02 and ID03).

Podophyllin has been successfully utilized as a treatment for CA patients, and it has been found to be effective in reducing superficial CA [13]. In randomized controlled trials, podophyllin treatment yields moderate clearance rates of 41%–47%, while recurrent rates were found to be around 25%–70% [13]. However, there are adverse effects of podophyllin, such as long-term toxicity, mutagenicity, and carcinogenesis [14]. Interestingly, *C. nutans* showed compelling effects in preclinical *in vitro* and *in vivo* studies, suggesting that it has multifaceted properties, involving in antiviral, anti-inflammation and antiproliferative activities [15]. Evidence from the meta-analysis of randomized controlled trial studies demonstrated that *C. nutans* cream is useful and has high efficacy in *Herpes genitalis* treatment [16]. In Thailand, 4% *C. nutans* cream (w/w) is commercially available, and it is recommended to apply 4 times daily [17]. However, there has been no medication recommendation for *C. nutans*-treated CA. This is the first study to evaluate the clinical effectiveness of *C. nutans* cream in a well-controlled clinical design. It was found that clearance rate of podophyllin treatment was similar to those observed by other clinical studies [18]. Interestingly, high clearance rate (84.85%) was found using *C. nutans* in LR-HPV-infected CA, but low clearance rate (50%) was found in HR-HPV-infected CA. These results suggested that *C. nutans* might be more effective in the clearance of LR-HPV infection than HR-HPV infection. In summary, podophyllin showed 74% reduction of HPV viral load in all patients, which was consistent with 97% CA lesion clearance, whereas *C. nutans* was able to reduce the HPV viral load for 42%, which was also consistent with 85% CA lesion clearance. These results were supported by our efficacy treatment analysis in that podophyllin cauterization was more effective than *C. nutans*. According to the *C. nutans* treatment, five of the six patients showed reduction in the HPV viral load in correlation with their CA lesion clearance. Only one patient (ID03) revealed high viral load even if the CA lesion was reduced, suggesting that *C. nutans* may not cure CA infected with HR-HPV.

Regardless of the efficacy treatment, there was a correlation between HPV genotyping and HPV viral load clearance. Podophyllin demonstrated significantly reduced viral load for both LR-HPV and HR-HPV, whereas *C. nutans* was found to reduce only LR-HPV viral load. Our results were consistent with other studies that podophyllin could effectively eliminate wart lesions from all HPV genotypes, resulting in no HPV detection [19]. As to the *C. nutans*, Sookmai et al. reported that *C. nutans* compounds could inhibit HPV infections in an *in vitro* study [6]. Therefore, it is suggested that podophyllin is an appropriate treatment for multiple HPV infections. On the other hand, *C. nutans* was less effective than podophyllin; hence, it is a suitable treatment for a single HPV infection and LR-HPV infection.

**Figure 3:**
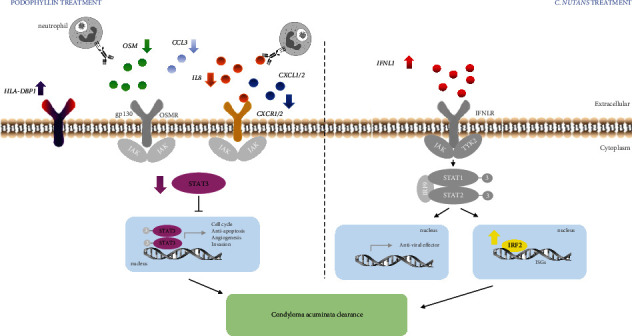
Proposed mechanism for podophyllin and *C. nutans* treatment. Various genes involving in inflammatory and immune response signaling were displayed. Gene cascade either be activated or inhibited, resulting in condyloma acuminata clearance.

The immune response in HPV-infected CA is considered to be the main determinant of CA disease and its associated immunomodulatory approaches, which have gained attention as promising strategies for CA treatment. Therefore, this is the first report on the mechanism of action associated with the immune response after podophyllin and *C. nutans* treatment. To define the target genes, differentially expressed genes were screened by the volcano plot method. From total of 23 genes, two genes were upregulated and 21 were downregulated after podophyllin treatment. Only *HLA-DPB1* was significantly upregulated, whereas *OSM, IL8, CXCL1, CXCL2*, and *CXCR2* were the five most significantly downregulated genes. Interestingly, *HLA-DPB1* was found to be upregulated after podophyllin treatment, which was a novel finding. *HLA-DPB1* belongs to *HLA* (human leukocyte antigen) class II, which is associated with a major histocompatibility complex in viral pathogenesis [20]. It is explained that *DRB1*^*∗*^*14*, a polymorphism of *HLA class II*, confers a protection against cervical cancer [21]. It is speculated in our findings that upregulation of the genes involving in immune response might allow a permissive environment for viral invasion and replication. Furthermore, the upregulation of *OSM* gene was statistically significant observed. The *OSM* gene, Oncostatin M, an *IL-6* family cytokine, helps to promote cell senescence and inhibits growth [22]. Previously, it was reported that *OSM* mRNA level was significantly upregulated in the *HPV16-E6* but downregulated in the HPV negative group [23]. This is consistent with our results, which indicated that *OSM* downregulation occurred with the podophyllin treatment. Therefore, podophyllin may inhibit or downregulate *OSM* gene due to the HPV infection clearance. Surprisingly, the expression levels of *IL8* and *CXCR2* genes were significantly downregulated. *IL8* is an important regulator of the innate immunity; it is also known as a potent proinflammatory cytokine that exerts its effects through binding to its G-protein-coupled receptor *CXCR1* and *CXCR2* [24]. Waugh et al. reported that inhibition of *IL8-CXCR1/CXCR2* has been shown to have therapeutic potential for a variety of solid tumors [25]. We suggested that *IL8* and *CXCR2* pathway may serve as a novel mechanism after podophyllin treatment. After *C. nutans* treatment, *IFNL1* and *IRF2* were found to be significantly upregulated, while none of other genes were found to be significantly downregulated. Interestingly, *IFNL1* or Interferon Lambda 1 stimulates clearance of norovirus infection independently of adaptive immune system, suggesting that innate immune responses may have a role in determining viral persistence [26]. Furthermore, *IFNL1* treatment was reported to decrease HSV-2 shedding from the vaginal mucosa in *in vivo* study [27]. Hence, it is speculated that upregulation of the *IFNL1* by *C. nutans* treatment, which would lead to the activation of antiviral state in epithelial cell, was a critical first-line defense against pathogenic viruses. Interferon regulatory factor 2*, IRF2,* has emerged as a complex mediator of gene expression and cell cycle control [28]. Lace et al. reported an increase in HPV-E7 expression, which could downregulate the *IRF2* promotor, displaying a complex negative feedback mechanism in response to signal transduction pathways [29]. This corresponds with our finding that upregulation of the *IRF2* gene would lead to elimination of the HPV infection. It is assumed that an increased immunosurveillance in CA lesions may allow the virus clearance with *C. nutans* treatment (Figure 3).

Surprisingly, 108 genes associated with inflammatory process were significantly downregulated with the podophyllin treatment (Figure S3), whereas none of those genes were found to be upregulated or downregulated after the *C. nutans* treatment (Figure S4). All of downregulated genes were found to be possibly associated with the proinflammatory cytokines, chemokines, and receptors synthetic pathways. In this study, *CCL3, CXCL2, CXCL1, IL8,* and *STAT3* were found to be the five most significantly downregulated genes. A significant downregulation of the genes was shown in response to a decreased expression of *CCL3*, *CXCL1*, and *CXCL2.* It is suggested that podophyllin might induce a negative feedback on the proinflammatory pathway, leading to a decrease in the size of lesions. Downregulation of *STAT3* after podophyllin treatment was also a novel finding. *STAT3*, a signal transducer and activator of transcription 3, modulates the transcription of the genes involved in the regulation of cell differentiation, proliferation, and immune responses. Frank et al. revealed that a selective inhibitor of *STAT3* signaling pathway could be used as an anticancer drug [30]. We suggested that downregulation of *STAT3* after podophyllin treatment may be associated with the inhibition of HPV proliferation due to lesion and viral load clearance (Figure 3).

In addition, the gene expression profiling associated with immune and inflammation panels of podophyllin and *C. nutans*-treated CA were compared. Some differences were found in immunomic profiling after podophyllin and *C. nutans* treatments. With podophyllin treatment, most genes involved in immune and inflammatory response were downregulated, whereas with the *C. nutans* treatment, those genes were upregulated. These results suggested that podophyllin and *C. nutans* may operate different mechanisms for HPV clearance. Downregulation of genes involving in immune and inflammatory response after podophyllin treatment suggested that its mechanism of action may not be correlated with the immune response; however, it may be related to previously described antiproliferative activity leading to CA clearance [31]. It is speculated that *C. nutans* might not inhibit HPV replication directly. It is demonstrated that *C. nutans* could stimulate the production of cytokines; hence, increase the immune response, thereby diminishing CA and decreasing virus erosion [5]. Furthermore, downregulation of inflammatory genes might suggest that HPV clearance mechanisms of both drugs may be associated with anti-inflammatory signaling pathway as described previously [4]. These findings suggested that podophyllin can exert more potent anti-inflammatory response than *C. nutans*. Alternatively, HPV clearance mechanism of *C. nutans* might not be involved in the inflammation signaling pathway. It is speculated that some other genes might play a crosstalk between the immune response and inflammation signaling pathways leading to HPV clearance by podophyllin or *C. nutans*. These shared molecular mechanisms may extend a potential target for a more effectively treatment of CA.

Nevertheless, there are some limitations of this study. Firstly, long-term follow-up more than 1 year was not completed. Secondly, a number of HPV-infected subjects was limited. Thirdly, heterogeneity leads to result uncertainty. Additionally, it is necessary to do further study on the molecular regulatory mechanism and other protein expression analysis of both drugs trials in order to identify therapeutic regimen. In turn, this may provide a guideline for the outcome on combination of these drugs for the CA treatment and indicate the underlying biological targets of CA in the future. However, this study might shed some light on applying *C. nutans* as an alternative treatment for HPV-related CA with less long-term toxic effect.

## 5. Conclusion

This study provides sufficient findings to demonstrate that the clinical efficacy and mechanism of action of podophyllin and *C. nutans* were, at least in part, due to reducing the inflammatory and modulating immune response signaling. Given the above findings, podophyllin possibly diminished HPV via both immune response and inflammatory signaling pathways, thereby allowing CA clearance. In contrast, *C. nutans* may promote the immune response while dampening inflammatory signaling, thereby given an overall tendency toward CA clearance.

## Figures and Tables

**Table 1:** Comparison of genotyping, lesion area and HPV viral load.

No.	Genotyping		Podophyllin treatment						*C. nutans* treatment					
			Size of wart (width × length × depth)			HPV viral load (HPV/cells)			Size of wart (width × length × depth)			HPV viral load (HPV/cells)		
	Low risk	High risk	Pretreatment (mm)	Posttreatment (mm)	% reduction	Pretreatment	Posttreatment	% reduction	Pretreatment (mm)	Posttreatment (mm)	% reduction	Pretreatment	Posttreatment	% reduction
01	6	—	20 × 5 × 3	0 × 0 × 0	100	1.46	0.00	100	10 × 5 × 3	0 × 0 × 0	100	0.16	0.02	98
02	11, 40	—	10 × 10 × 3	3 × 3 × 1	97	1.96	0.41	79	20 × 5 × 5	5 × 3 × 3	91	1.23	0.71	42
03	11, 81	45, 58	30 × 20 × 10^a^	20 × 16 × 8^b^	70	1.77	1.07	40	30 × 20 × 10^c^	25 × 20 × 10^d^	50	0.87	3.64	−318
04	6	—	3 × 3 × 2	1 × 1 × 0.5	97	2.13	0.65	69	2 × 3 × 2	1.5 × 1 × 1	82	2.19	1.17	47
05	6	—	2 × 3 × 2	0 × 0 × 0	100	1.39	0.13	91	3 × 5 × 3	2 × 4 × 1	87	1.90	1.37	28
06	11	—	2 × 2 × 2	1 × 1 × 1	87	1.76	0.34	81	3 × 5 × 3	2 × 3 × 2	73	1.74	0.57	67
Median (min, max)					97 (70, 100)	1.77 (1.39, 2.13)	0.38 (0.00, 1.07)	74 (40, 91)	Median (min, max)		85 (50, 100)	1.49 (0.87, 2.19)	0.94 (0.02, 3.64)	42 (−318, 67)

^a^14 lesions; ^b^10 lesions; ^c^15 lesions; ^d^9 lesions.

## Data Availability

The data used to support the findings of this study are included within the supplementary information files.

## Supplementary Materials

Supplementary Materials Table S1: oligonucleotide primers and thermal cycling condition of HPV and *β*-globin gene. Table S2: top 20 differentially expressed gene in pre- and postpodophyllin treatments. Table S3: top 20 differentially expressed gene of pre- and post-*C. nutans* treatment in immune panel. Table S4: top 20 differentially expressed genes in inflammatory panel from pre- and postpodophyllin treatments. Table S5: top 20 differentially expressed gene of pre- and post-*C. nutans* treatment in inflammatory panel. Figure S1: volcano plot showing differentially expressed genes in the immune panel from podophyllin treatment using NanoString Technology. Figure S2: volcano plot showing differentially expressed genes in the immune panel from *C. nutans* treatment using NanoString Technology. Figure S3: the volcano plot showing differentially expressed genes in the inflammatory panel from podophyllin treatment. Figure S4: volcano plot showing differentially expressed genes in the inflammatory panel from *C. nutans* treatment using NanoString Technology. File S1: (A) Clinical manifestation. (B) Agarose gel electrophoretic pattern of pre- and postdrug treatments. File S2: (A) Venn diagram of the number of gene expressions in the immune panels of the podophyllin and *C. nutans* treatment groups. (B) Top 20 differentially expressed genes from the podophyllin and *C. nutans* treated 24 CA samples in immune panel using NanoString Technology.
