# Supplementary material for: Expression Pattern of Genes in Condyloma Acuminata Treated with Clinacanthus nutans Lindau Cream versus Podophyllin
Source: Evid Based Complement Alternat Med. 2021 Sep 17;2021:5579520. doi: 10.1155/2021/5579520 (PMC8463201; doi:10.1155/2021/5579520)
Supplement: Supplementary Materials — Table S1: oligonucleotide primers and thermal cycling condition of HPV and β-globin gene. Table S2: top 20 differentially expressed gene in pre- and postpodophyllin treatments. Table S3: top 20 differentially expressed gene of pre- and post-C. nutans treatment in immune panel. Table S4: top 20 differentially expressed genes in inflammatory panel from pre- and postpodophyllin treatments. Table S5: top 20 differentially expressed gene of pre- and post-C. nutans treatment in inflammatory panel. Figure S1: volcano plot showing differentially expressed genes in the immune panel from podophyllin treatment using NanoString Technology. Figure S2: volcano plot showing differentially expressed genes in the immune panel from C. nutans treatment using NanoString Technology. Figure S3: the volcano plot showing differentially expressed genes in the inflammatory panel from podophyllin treatment. Figure S4: volcano plot showing differentially expressed genes in the inflammatory panel from C. nutans treatment using NanoString Technology. File S1: (A) Clinical manifestation. (B) Agarose gel electrophoretic pattern of pre- and postdrug treatments. File S2: (A) Venn diagram of the number of gene expressions in the immune panels of the podophyllin and C. nutans treatment groups. (B) Top 20 differentially expressed genes from the podophyllin and C. nutans treated 24 CA samples in immune panel using NanoString Technology. [file 5579520.f1.zip › 5579520.f1/File S1 (1).pdf]

File S1

(A) Clinical manifestation. Condyloma acuminata on shaft, neck and glans of penis was treated with podophyllin or *C.nutans* for 4 weeks with subsequent reduction in CA lesion.

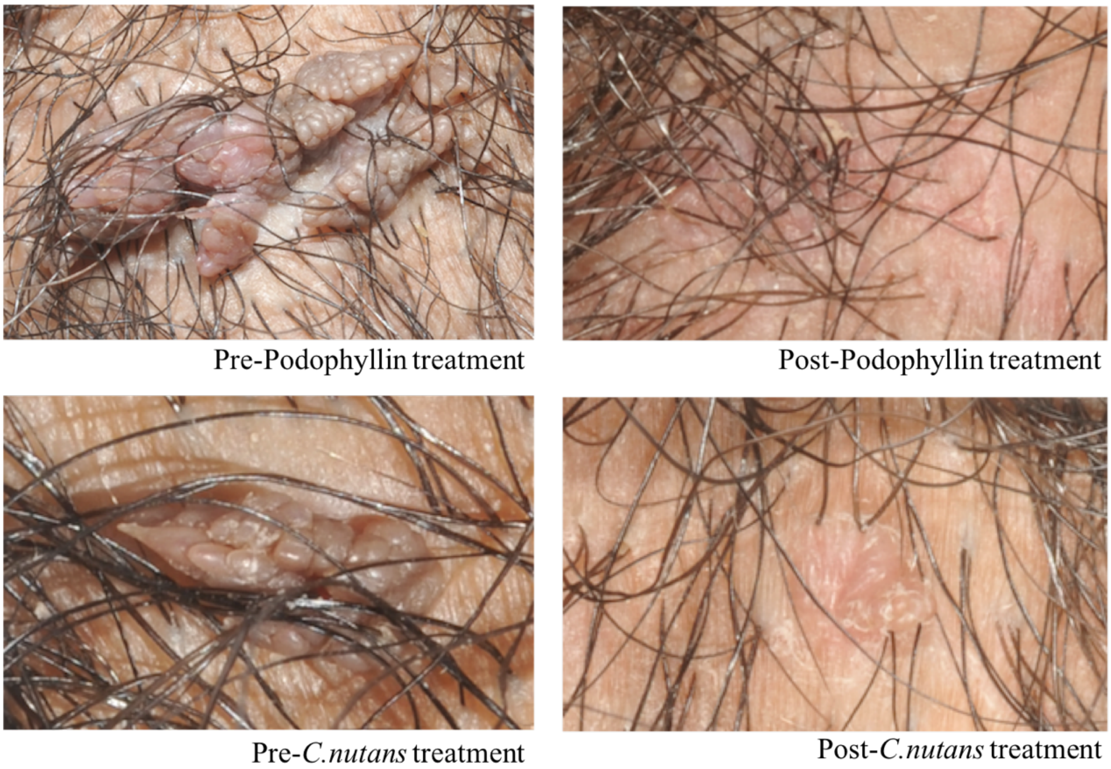

File S1_Figure A1 Photodocumentation of sample ID01

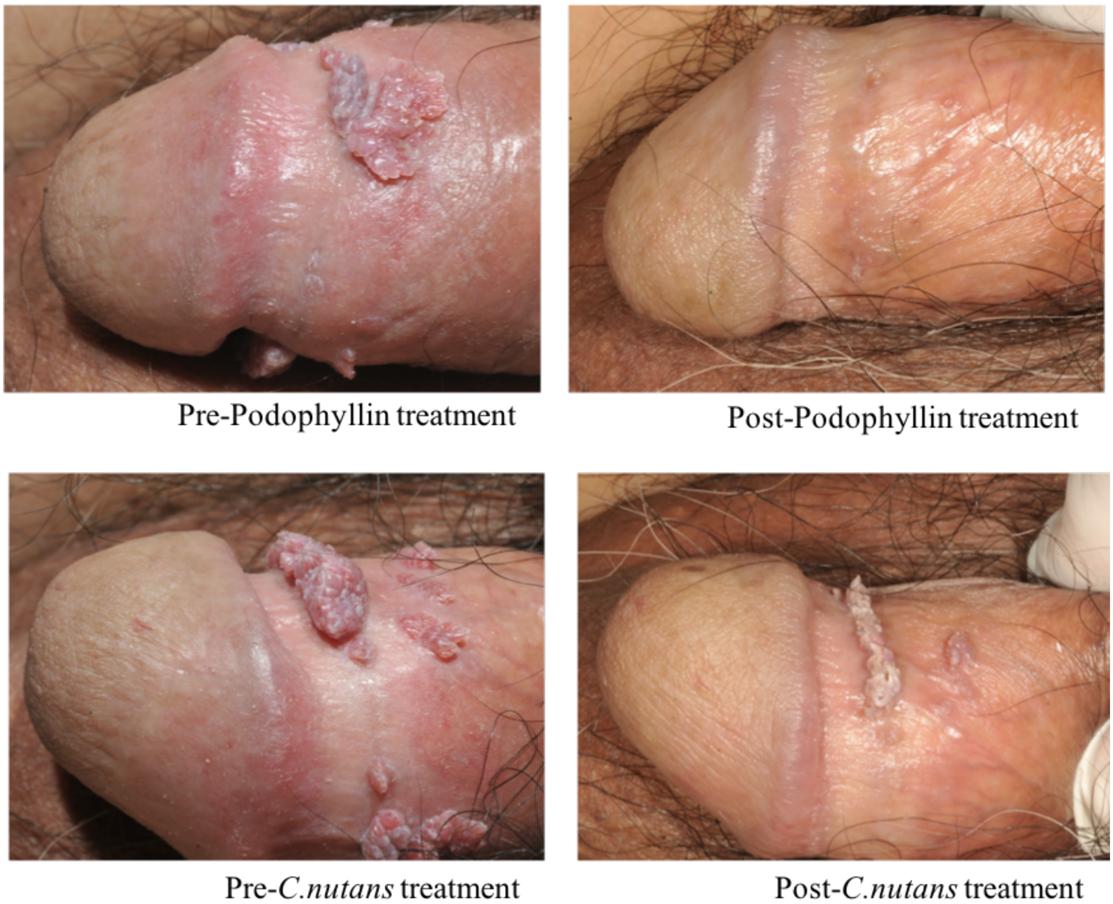

File S1_Figure A2 Photodocumentation of sample ID02

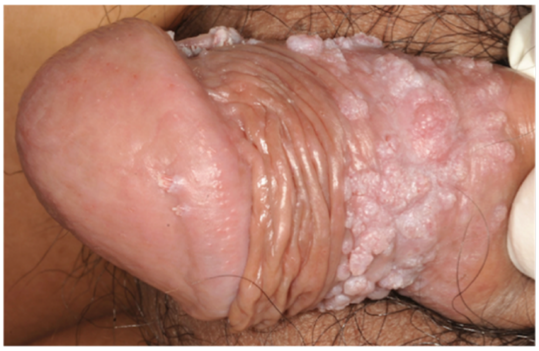

Pre-Podophyllin treatment

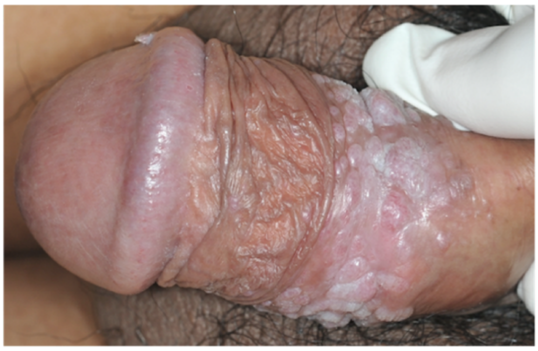

Post-Podophyllin treatment

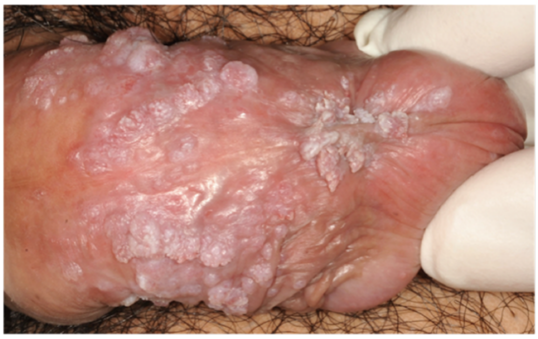

Pre-*C.nutans* treatment

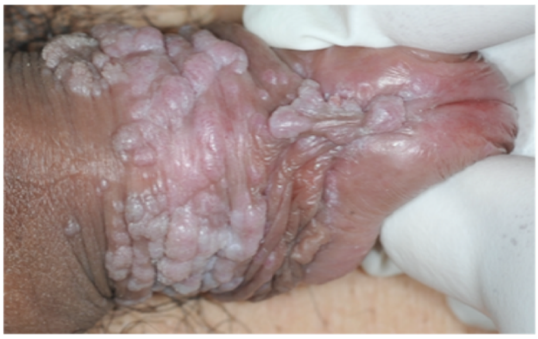

Post-*C.nutans* treatment

File S1_Figure A3 Photodocumentation of sample ID03

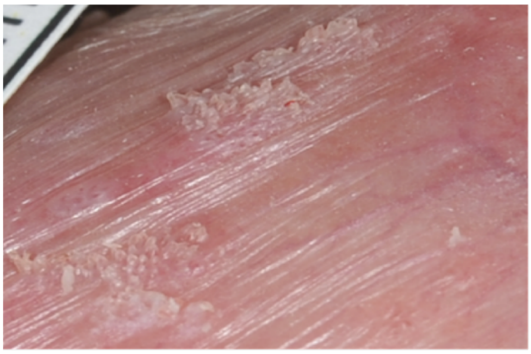

Pre-Podophyllin treatment

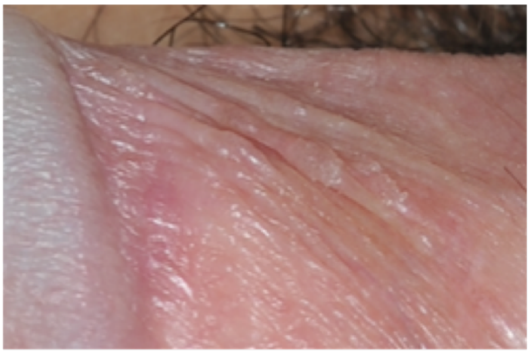

Post-Podophyllin treatment

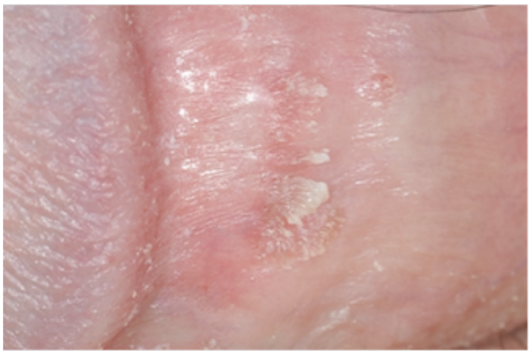

Pre-*C.nutans* treatment

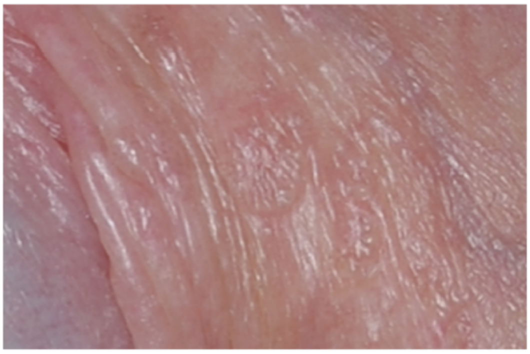

Post-*C.nutans* treatment

File S1_Figure A4 Photodocumentation of sample ID04

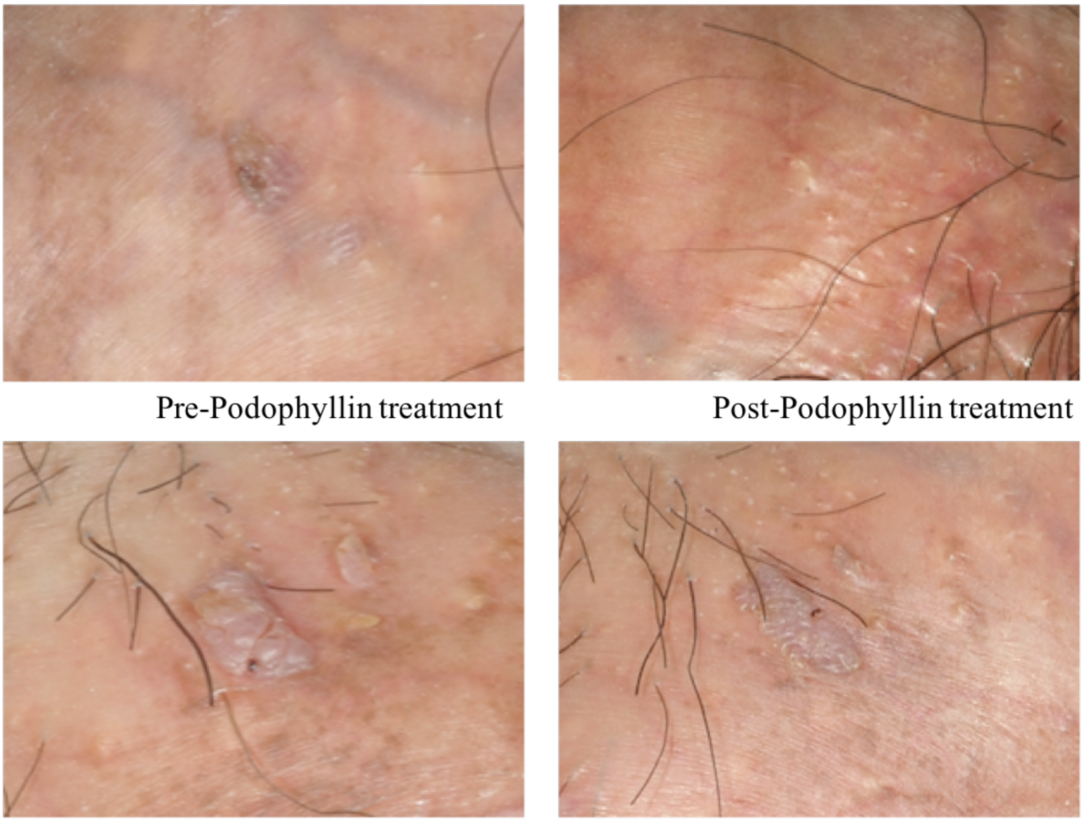

Pre-Podophyllin treatment

Post-Podophyllin treatment

Pre-*C.nutans* treatment

Post-*C.nutans* treatment

File S1_Figure A5 Photodocumentation of sample ID05

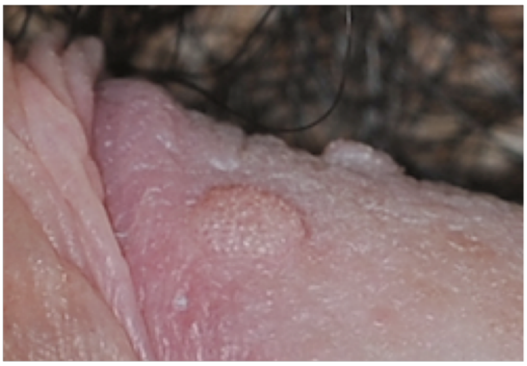

Pre-Podophyllin treatment

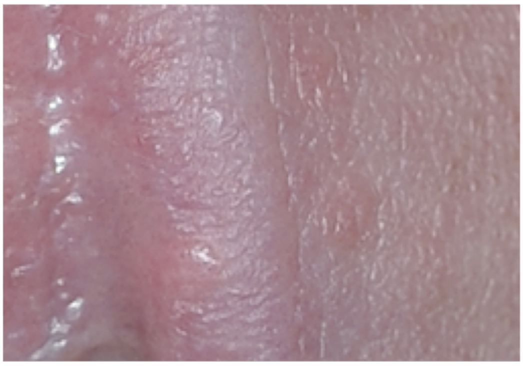

Post-Podophyllin treatment

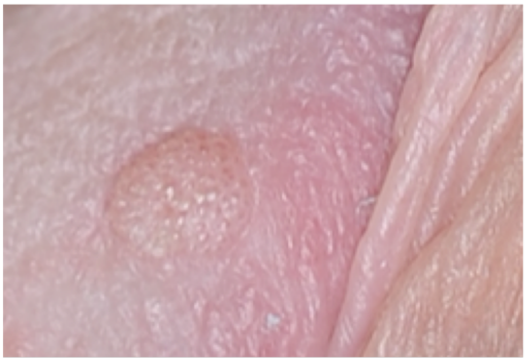

Pre-*C.nutans* treatment

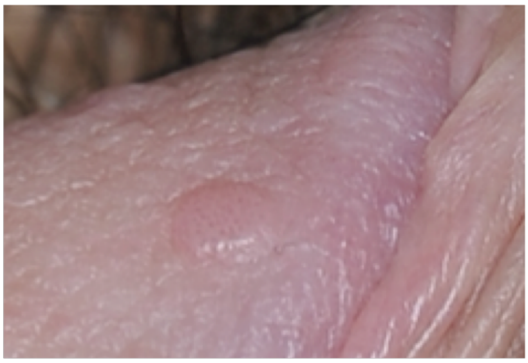

Post-*C.nutans* treatment

File S1_Figure A6 Photodocumentation of sample ID06

(B) Agarose gel electrophoretic pattern. Analysis of the PCR-amplified HPV products of pre- and post- podophyllin treatments in comparison with pre- and post- *C. nutans* treatments from condyloma acuminata samples.

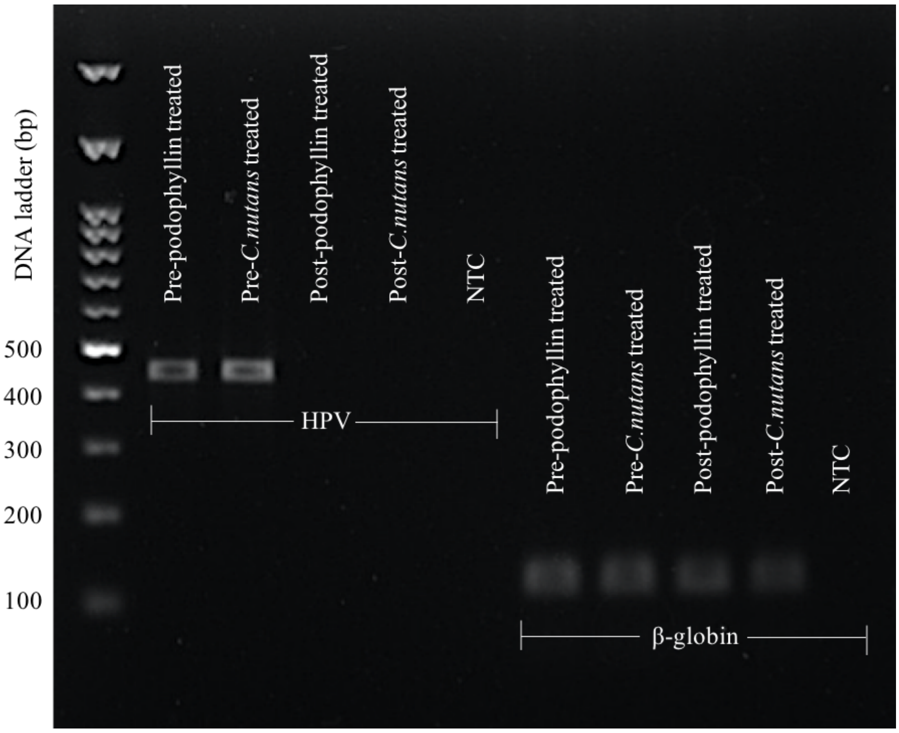

File S1_Figure B1 1.5% Agarose gel electrophoretic pattern of PCR-amplified products from sample ID01.

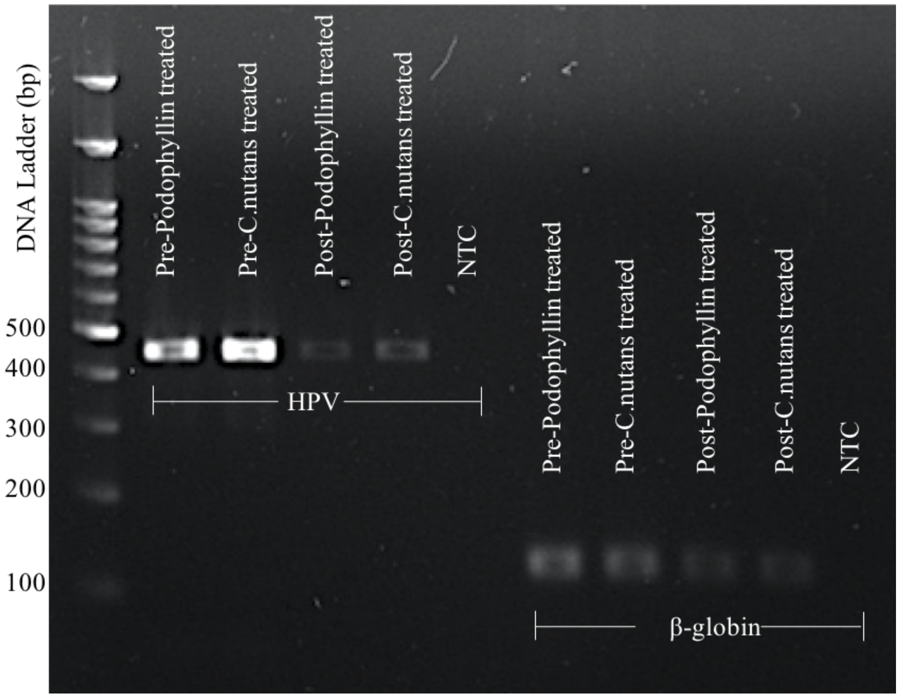

File S1_Figure B2 1.5% Agarose gel electrophoretic pattern of PCR-amplified products from sample ID02.

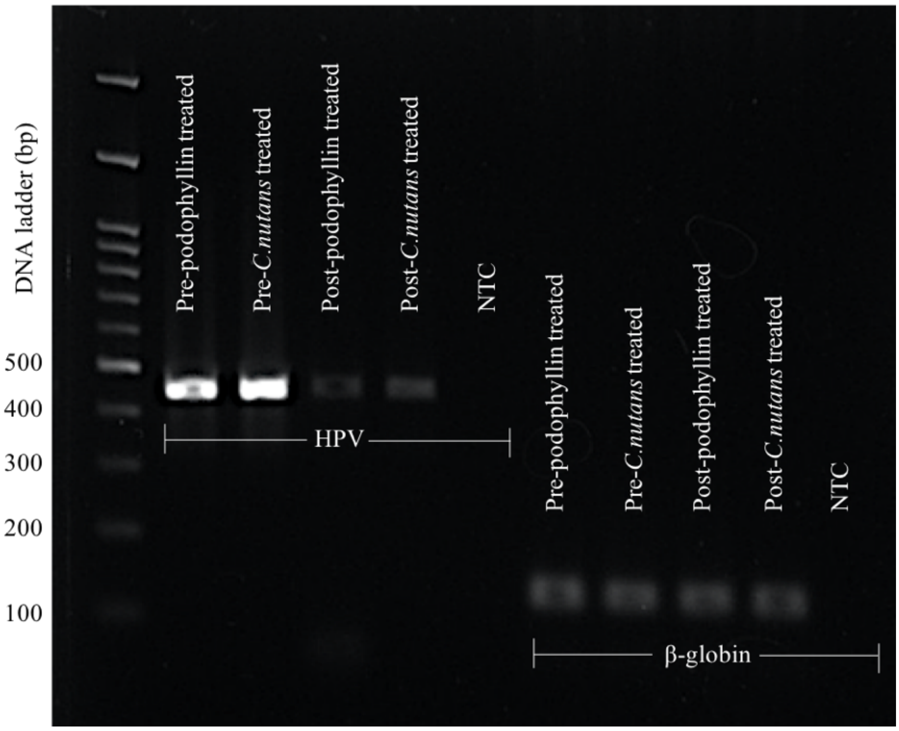

File S1_Figure B3 1.5% Agarose gel electrophoretic pattern of PCR-amplified products from sample ID03.

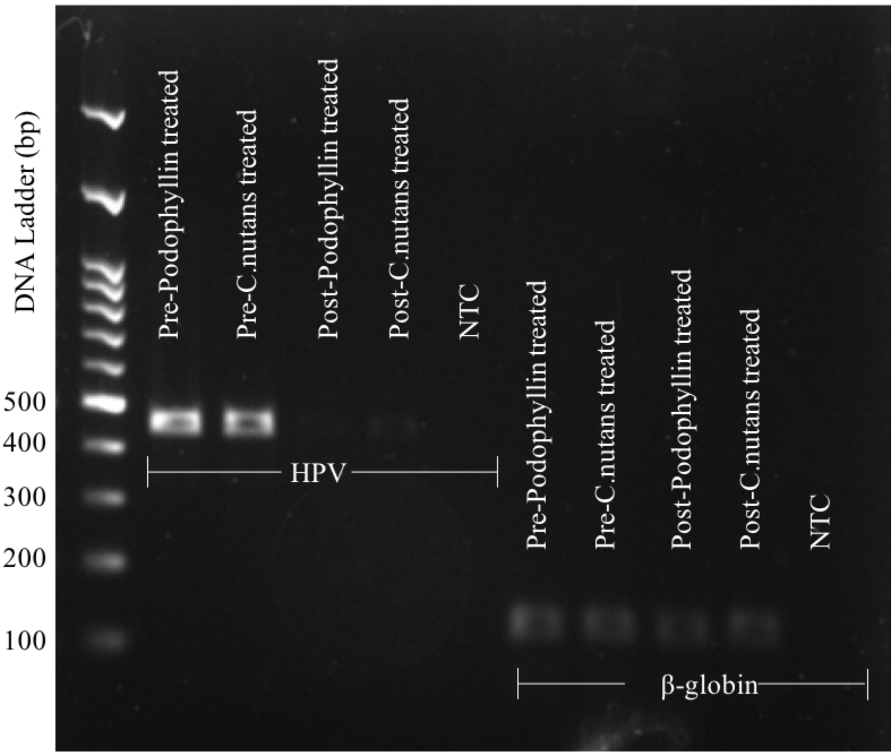

File S1_Figure B4 1.5% Agarose gel electrophoretic pattern of PCR-amplified products from sample ID04.

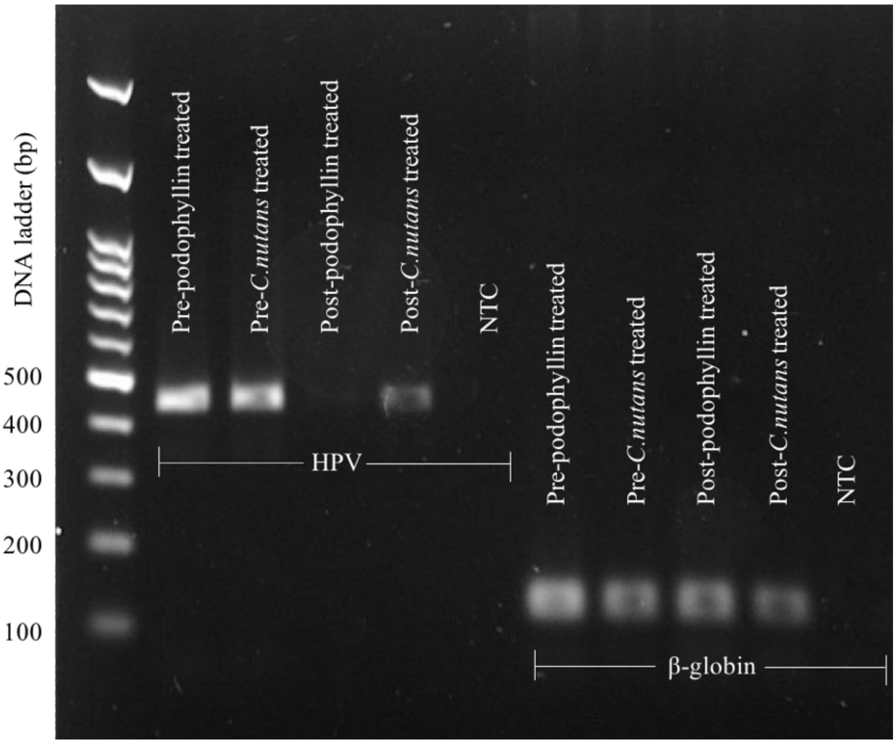

File S1_Figure B5 1.5% Agarose gel electrophoretic pattern of PCR-amplified products from sample ID05.

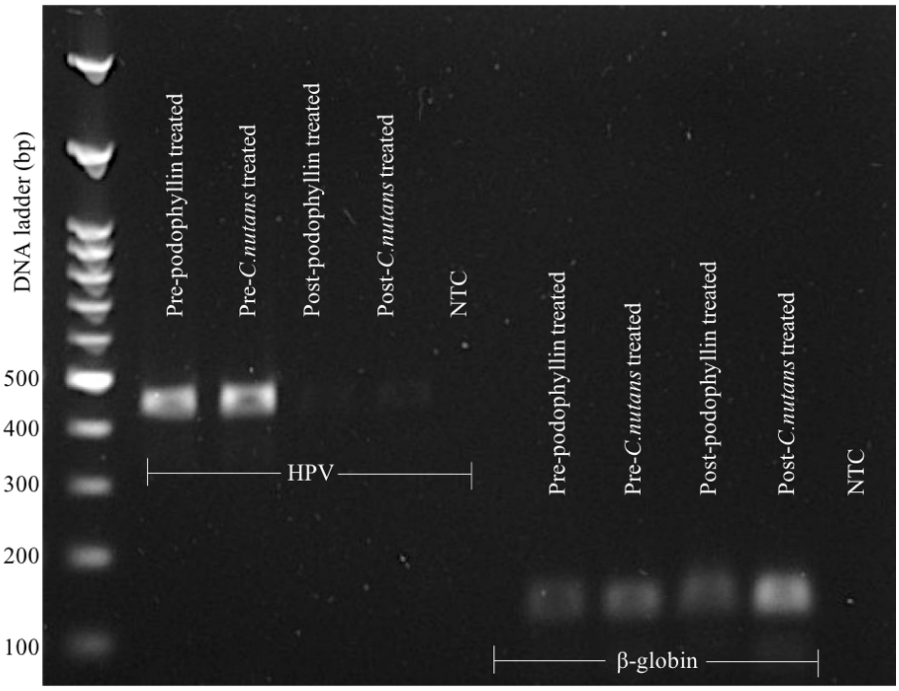

File S1_Figure B6 1.5% Agarose gel electrophoretic pattern of PCR-amplified products from sample ID06.
