# Supplementary material for: Expression Pattern of Genes in Condyloma Acuminata Treated with Clinacanthus nutans Lindau Cream versus Podophyllin
Source: Evid Based Complement Alternat Med. 2021 Sep 17;2021:5579520. doi: 10.1155/2021/5579520 (PMC8463201; doi:10.1155/2021/5579520)
Supplement: Supplementary Materials — Table S1: oligonucleotide primers and thermal cycling condition of HPV and β-globin gene. Table S2: top 20 differentially expressed gene in pre- and postpodophyllin treatments. Table S3: top 20 differentially expressed gene of pre- and post-C. nutans treatment in immune panel. Table S4: top 20 differentially expressed genes in inflammatory panel from pre- and postpodophyllin treatments. Table S5: top 20 differentially expressed gene of pre- and post-C. nutans treatment in inflammatory panel. Figure S1: volcano plot showing differentially expressed genes in the immune panel from podophyllin treatment using NanoString Technology. Figure S2: volcano plot showing differentially expressed genes in the immune panel from C. nutans treatment using NanoString Technology. Figure S3: the volcano plot showing differentially expressed genes in the inflammatory panel from podophyllin treatment. Figure S4: volcano plot showing differentially expressed genes in the inflammatory panel from C. nutans treatment using NanoString Technology. File S1: (A) Clinical manifestation. (B) Agarose gel electrophoretic pattern of pre- and postdrug treatments. File S2: (A) Venn diagram of the number of gene expressions in the immune panels of the podophyllin and C. nutans treatment groups. (B) Top 20 differentially expressed genes from the podophyllin and C. nutans treated 24 CA samples in immune panel using NanoString Technology. [file 5579520.f1.zip › 5579520.f1/Supplementary Description.docx]

## Supplementary Materials

**Table S1:** Oligonucleotide Primers and Thermal cycling condition of HPV and β-globin gene

**Table S2:** Top 20 differentially expressed gene in pre-and post podophyllin treatments.

**Table S3:** Top 20 differentially expressed gene of pre-and post *C. nutans* treatment in immune panel.

**Table S4:** Top 20 differentially expressed genes in inflammatory panel from pre-and post- podophyllin treatments.

**Table S5:** Top 20 differentially expressed gene of pre-and post *C. nutans* treatment in inflammatory panel.

**Figure S1:** Volcano plot showing differentially expressed genes in the immune panel from podophyllin treatment using NanoString Technology.

**Figure S2:** Volcano plot showing differentially expressed genes in the immune panel from *C. nutans* treatment using NanoString Technology.

**Figure S3:** The volcano plot showing differentially expressed genes in the inflammatory panel from podophyllin treatment.

**Figure S4:** Volcano plot showing differentially expressed genes in the inflammatory panel from *C. nutans* treatment using NanoString Technology.

**File S1:** (A) Clinical manifestation. (B) Agarose gel electrophoretic pattern of pre- and post- drug treatments.

**File S2:** (A) Venn diagram of the number of gene expressions in the immune panels of the podophyllin and *C. nutans* treatment groups. (B) Top 20 differentially expressed genes from the podophyllin and *C. nutans* treated 24 CA samples in immune panel using NanoString Technology.
