# Supplementary material for: Expression Pattern of Genes in Condyloma Acuminata Treated with Clinacanthus nutans Lindau Cream versus Podophyllin
Source: Evid Based Complement Alternat Med. 2021 Sep 17;2021:5579520. doi: 10.1155/2021/5579520 (PMC8463201; doi:10.1155/2021/5579520)
Supplement: Supplementary Materials — Table S1: oligonucleotide primers and thermal cycling condition of HPV and β-globin gene. Table S2: top 20 differentially expressed gene in pre- and postpodophyllin treatments. Table S3: top 20 differentially expressed gene of pre- and post-C. nutans treatment in immune panel. Table S4: top 20 differentially expressed genes in inflammatory panel from pre- and postpodophyllin treatments. Table S5: top 20 differentially expressed gene of pre- and post-C. nutans treatment in inflammatory panel. Figure S1: volcano plot showing differentially expressed genes in the immune panel from podophyllin treatment using NanoString Technology. Figure S2: volcano plot showing differentially expressed genes in the immune panel from C. nutans treatment using NanoString Technology. Figure S3: the volcano plot showing differentially expressed genes in the inflammatory panel from podophyllin treatment. Figure S4: volcano plot showing differentially expressed genes in the inflammatory panel from C. nutans treatment using NanoString Technology. File S1: (A) Clinical manifestation. (B) Agarose gel electrophoretic pattern of pre- and postdrug treatments. File S2: (A) Venn diagram of the number of gene expressions in the immune panels of the podophyllin and C. nutans treatment groups. (B) Top 20 differentially expressed genes from the podophyllin and C. nutans treated 24 CA samples in immune panel using NanoString Technology. [file 5579520.f1.zip › 5579520.f1/Table S2.pdf]

Table S2

Top 20 differentially expressed gene in pre-and post podophyllin treatments. (Genes were ordered by significant differentially expressed gene)

Gene	Log_2 Fold change	p-value Adjusted	Gene Set
HLA-DPB1	2.33	0.02	Antigen Processing
OSM	-6.77	0.02	Cell Functions
IL8	-6.73	0.02	Chemokines, Cytokines, Interleukins, Pathogen Defense, Regulation
CXCL2	-6.73	0.02	Chemokines, Regulation
CXCR2	-6.84	0.02	Chemokines, Regulation
CXCL1	-6.6	0.02	Chemokines, Regulation
CEBPB	-6.46	0.02	Cell Functions
CCL3L1	-6.93	0.02	Cytokines
IFNL1	3.19	0.03	Cytokines, Interleukins
IL1RN	-4.45	0.03	Cytokines, Interleukins
CTSS	-5.57	0.03	Cytotoxic
LCP1	-4.73	0.03	Leukocyte Functions, Macrophage Functions, T-Cell Functions
S100A12	-5.06	0.03	Pathogen Defense
IFITM2	-5.61	0.03	Regulation
IFITM1	-6.48	0.03	Regulation
TREM1	-7.01	0.03	Regulation
HLA-E	-5.17	0.03	Regulation
TANK	-4.77	0.03	TLR
TNFRSF10C	-4.33	0.03	TNF Superfamily
TNFAIP3	-5.43	0.03	TNF Superfamily

Adjust p-value was obtained from the adjusted p-value of FDR correction by Benjamini-Hochberg method
