# Supplementary material for: Expression Pattern of Genes in Condyloma Acuminata Treated with Clinacanthus nutans Lindau Cream versus Podophyllin
Source: Evid Based Complement Alternat Med. 2021 Sep 17;2021:5579520. doi: 10.1155/2021/5579520 (PMC8463201; doi:10.1155/2021/5579520)
Supplement: Supplementary Materials — Table S1: oligonucleotide primers and thermal cycling condition of HPV and β-globin gene. Table S2: top 20 differentially expressed gene in pre- and postpodophyllin treatments. Table S3: top 20 differentially expressed gene of pre- and post-C. nutans treatment in immune panel. Table S4: top 20 differentially expressed genes in inflammatory panel from pre- and postpodophyllin treatments. Table S5: top 20 differentially expressed gene of pre- and post-C. nutans treatment in inflammatory panel. Figure S1: volcano plot showing differentially expressed genes in the immune panel from podophyllin treatment using NanoString Technology. Figure S2: volcano plot showing differentially expressed genes in the immune panel from C. nutans treatment using NanoString Technology. Figure S3: the volcano plot showing differentially expressed genes in the inflammatory panel from podophyllin treatment. Figure S4: volcano plot showing differentially expressed genes in the inflammatory panel from C. nutans treatment using NanoString Technology. File S1: (A) Clinical manifestation. (B) Agarose gel electrophoretic pattern of pre- and postdrug treatments. File S2: (A) Venn diagram of the number of gene expressions in the immune panels of the podophyllin and C. nutans treatment groups. (B) Top 20 differentially expressed genes from the podophyllin and C. nutans treated 24 CA samples in immune panel using NanoString Technology. [file 5579520.f1.zip › 5579520.f1/Table S3.pdf]

Table S3

Top 20 differentially expressed gene of pre-and post *C.nutans* treatment in immune panel. (Genes were ordered by significant differentially expressed gene)

Gene	Log_2 fold change	Adjusted p-value[a]	Gene Set
IFNL1*	4.49	0.02	Cytokines, Interleukins
IRF2*	3.01	0.05	Chemokines, Regulation
KIR_Inhibiting_Subgroup_2	3.54	0.06	Cell Functions, NK Cell Functions
NT5E	2.62	0.15	Transporter Functions
KIR_Inhibiting_Subgroup_1	3.69	0.15	Cell Functions, NK Cell Functions, Regulation
CD14	-5.89	0.36	Macrophage Function
S100A12	-6.29	0.36	Pathogen Defense
CXCL2	-8.1	0.36	Chemokines, Regulation
CD79B	2.79	0.36	B-Cell Functions
CR2	3.05	0.36	B-Cell Functions
CD74	-5.64	0.36	Antigen Processing
CXCL3	-5.09	0.36	Chemokines, Regulation
CD53	-4.77	0.36	T-cell function
TANK	-5.79	0.36	TLR
CXCR4	-4.67	0.36	Cell Cycle, Cell Functions, Chemokines, Regulation
TLR6	-3.96	0.36	TLR
TNFRSF10C	-4.54	0.36	TNF Superfamily
CXCL1	-2.94	0.36	Chemokines, Regulation
CD59	-4.43	0.36	Cytotoxicity
NCF4	-4.36	0.36	Cell functions

[a] Adjust p-value was obtained from the adjusted p-value of FDR correction by Benjamini-Hochberg method

* Significant difference, adjusted $p \leq 0.05$
