# Supplementary material for: Expression Pattern of Genes in Condyloma Acuminata Treated with Clinacanthus nutans Lindau Cream versus Podophyllin
Source: Evid Based Complement Alternat Med. 2021 Sep 17;2021:5579520. doi: 10.1155/2021/5579520 (PMC8463201; doi:10.1155/2021/5579520)
Supplement: Supplementary Materials — Table S1: oligonucleotide primers and thermal cycling condition of HPV and β-globin gene. Table S2: top 20 differentially expressed gene in pre- and postpodophyllin treatments. Table S3: top 20 differentially expressed gene of pre- and post-C. nutans treatment in immune panel. Table S4: top 20 differentially expressed genes in inflammatory panel from pre- and postpodophyllin treatments. Table S5: top 20 differentially expressed gene of pre- and post-C. nutans treatment in inflammatory panel. Figure S1: volcano plot showing differentially expressed genes in the immune panel from podophyllin treatment using NanoString Technology. Figure S2: volcano plot showing differentially expressed genes in the immune panel from C. nutans treatment using NanoString Technology. Figure S3: the volcano plot showing differentially expressed genes in the inflammatory panel from podophyllin treatment. Figure S4: volcano plot showing differentially expressed genes in the inflammatory panel from C. nutans treatment using NanoString Technology. File S1: (A) Clinical manifestation. (B) Agarose gel electrophoretic pattern of pre- and postdrug treatments. File S2: (A) Venn diagram of the number of gene expressions in the immune panels of the podophyllin and C. nutans treatment groups. (B) Top 20 differentially expressed genes from the podophyllin and C. nutans treated 24 CA samples in immune panel using NanoString Technology. [file 5579520.f1.zip › 5579520.f1/Table S4.pdf]

Table S4

Top 20 differentially expressed genes in inflammatory panel from pre-and post-podophyllin treatments. (Genes were ordered by significant differentially expressed gene)

	Log_2 fold change	Adjusted p.value	Gene sets
CXCL2	-4.64	0.01	Behavior, Cell Fraction, Chemokine Activity, Defense Response, Extracellular Region, Extracellular Region Part, Extracellular Space, G Protein Coupled Receptor Binding, Inflammatory Response, Locomotory Behavior, Response To Chemical Stimulus, Response To External Stimulus, Response To Stress, Response To Wounding, Soluble Fraction
IL8	-7.17	0.01	Cell Activation, Cell Surface Receptor Linked Signal Transduction Go 0007166, Extracellular Region, Extracellular Region Part, Extracellular Space, Positive Regulation Of Response To Stimulus, Reproductive Process, Signal Transduction, System Development
CEBPB	-5.7	0.01	Biopolymer Metabolic Process, Defense Response, Immune Response, Immune System Process, Inflammatory Response, Nucleobasenucleosidenucleotide And Nucleic Acid Metabolic Process, Nucleus, Response To External Stimulus, Response To Stress, Rna Biosynthetic Process, Rna Metabolic Process, Transcription, Transcription Dna Dependent
TYROBP	-5.8	0.01	Defense Response, Integral To Membrane, Integral To Plasma Membrane, Intracellular Signaling Cascade, Intrinsic To Membrane, Intrinsic To Plasma Membrane, Membrane, Membrane Part, Plasma Membrane, Plasma Membrane Part, Signal Transduction
CFL1	-5.3	0.01	Actin Filament Based Process, Cell Development, Cytoplasm, Negative Regulation Of Apoptosis, Negative Regulation Of Cellular Process, Nucleus, Organelle Organization And Biogenesis, Programmed Cell Death, Ras Protein Signal Transduction, Regulation Of Developmental Process, Signal Transduction
STAT3	-5.36	0.01	Biopolymer Metabolic Process, Cytoplasm, Jak Stat Cascade, Negative Regulation Of Cellular Metabolic Process, Negative Regulation Of Nucleobasenucleosidenucleotide And Nucleic Acid Metabolic Process, Nervous System Development, Nucleus, Rna Metabolic Process, Signal Transduction, System Development
FOS	-4.19	0.01	Biopolymer Metabolic Process, Defense Response, Dna Metabolic Process, Inflammatory Response, Nucleobasenucleosidenucleotide And Nucleic Acid Metabolic Process, Nucleus, Rna Biosynthetic Process, Rna Metabolic Process, Transcription Dna Dependent
TGFB1	-3.16	0.01	Dna Metabolic Process, Extracellular Region, Extracellular Region Part, Extracellular Space, Muscle Development, Regulation Of Biological Quality, Rna Metabolic Process
CCL3	-4.84	0.01	Cell Fraction, Cell Surface Receptor Linked Signal Transduction Go 0007166, Establishment Of Cellular Localization, Establishment Of Localization, Organelle Organization And Biogenesis, Regulation Of Biological Quality, Reproductive Process, Signal Transduction, Soluble Fraction

Top 20 differentially expressed genes in inflammatory panel from pre-and post-podophyllin treatments. (cont.) (Genes were ordered by significant differentially expressed gene)

	Log_2 fold change	Adjusted p.value	Gene sets
IL18RAP	-3.07	0.01	Cell Surface Receptor Linked Signal Transduction Go 0007166, Defense Response, Inflammatory Response, Response To External Stimulus, Response To Stress, Response To Wounding, Signal Transduction
PTGS2	-3.72	0.01	Cytoplasm, Nucleus, Regulation Of Biological Quality
CXCL1	-2.35	0.01	Actin Filament Based Process, Behavior, Cell Surface Receptor Linked Signal Transduction Go 0007166, Defense Response, Extracellular Region, Extracellular Region Part, Extracellular Space, Negative Regulation Of Cellular Process, Nervous System Development, Organelle Organization And Biogenesis, Signal Transduction, System Development
GNAQ	-2.96	0.01	Cell Surface Receptor Linked Signal Transduction Go 0007166, Cytoplasm, Membrane, Plasma Membrane, Regulation Of Biological Quality, Regulation Of Molecular Function, Signal Transduction
TNFSF14	-2.86	0.01	Cell Development, Establishment Of Cellular Localization, Establishment Of Localization, I Kappab Kinase Nf Kappab Cascade, Programmed Cell Death, Signal Transduction
CCL20	-4.69	0.01	Behavior, Cell Cell Signaling, Defense Response, Extracellular Region, Extracellular Region Part, Extracellular Space, Immune Response, Immune System Process, Inflammatory Response, Locomotory Behavior, Response To Chemical Stimulus, Response To External Stimulus, Response To Stress, Response To Wounding, Signal Transduction
MAFG	-2.74	0.01	Hemostasis
MAX	-2.8	0.01	Biopolymer Metabolic Process, Nucleobasenucleosidenucleotide And Nucleic Acid Metabolic Process, Rna Biosynthetic Process, Rna Metabolic Process, Transcription Dna Dependent, Transcription Factor Activity, Transcription From Rna Polymerase Ii Promoter
IL21	-2.89	0.01	Cell Activation, Cell Development, Positive Regulation Of Cell Proliferation, Positive Regulation Of Immune System Process
C1QB	-2.87	0.01	Extracellular Region, Extracellular Region Part, Extracellular Space, Macromolecular Complex, Protein Complex
HIF1A	-5.08	0.02	Intracellular Organelle Part, Macromolecular Complex, Membrane Enclosed Lumen, Nuclear Part, Nucleus, Organelle Lumen, Organelle Part, Protein Complex, Regulation Of Biological Quality, Rna Metabolic Process
