# Supplementary material for: Expression Pattern of Genes in Condyloma Acuminata Treated with Clinacanthus nutans Lindau Cream versus Podophyllin
Source: Evid Based Complement Alternat Med. 2021 Sep 17;2021:5579520. doi: 10.1155/2021/5579520 (PMC8463201; doi:10.1155/2021/5579520)
Supplement: Supplementary Materials — Table S1: oligonucleotide primers and thermal cycling condition of HPV and β-globin gene. Table S2: top 20 differentially expressed gene in pre- and postpodophyllin treatments. Table S3: top 20 differentially expressed gene of pre- and post-C. nutans treatment in immune panel. Table S4: top 20 differentially expressed genes in inflammatory panel from pre- and postpodophyllin treatments. Table S5: top 20 differentially expressed gene of pre- and post-C. nutans treatment in inflammatory panel. Figure S1: volcano plot showing differentially expressed genes in the immune panel from podophyllin treatment using NanoString Technology. Figure S2: volcano plot showing differentially expressed genes in the immune panel from C. nutans treatment using NanoString Technology. Figure S3: the volcano plot showing differentially expressed genes in the inflammatory panel from podophyllin treatment. Figure S4: volcano plot showing differentially expressed genes in the inflammatory panel from C. nutans treatment using NanoString Technology. File S1: (A) Clinical manifestation. (B) Agarose gel electrophoretic pattern of pre- and postdrug treatments. File S2: (A) Venn diagram of the number of gene expressions in the immune panels of the podophyllin and C. nutans treatment groups. (B) Top 20 differentially expressed genes from the podophyllin and C. nutans treated 24 CA samples in immune panel using NanoString Technology. [file 5579520.f1.zip › 5579520.f1/Table S5.pdf]

Table S5

Top 20 differentially expressed gene of pre-and post *C.nutans* treatment in inflammatory panel. (Genes were ordered by significant differentially expressed gene)

	Log_2 Fold Change	Adjusted P-value	Gene Set
CXCL2	-3.31	0.10	Behavior, Cell Fraction, Chemokine Activity, Defense Response, Extracellular Region, Extracellular Region Part, Extracellular Space, G Protein Coupled Receptor Binding, Inflammatory Response, Locomotory Behavior, Response To Chemical Stimulus, Response To External Stimulus, Response To Stress, Response To Wounding, Soluble Fraction
TNFSF14	-3.12	0.10	Cell Development, Establishment Of Cellular Localization, Establishment Of Localization, I Kappab Kinase Nf Kappab Cascade, Programmed Cell Death, Signal Transduction
IL18RAP	-3.07	0.10	Cell Surface Receptor Linked Signal Transduction Go 0007166, Defense Response, Inflammatory Response, Response To External Stimulus, Response To Stress, Response To Wounding, Signal Transduction
CCL3	-5.06	0.10	Cell Fraction, Cell Surface Receptor Linked Signal Transduction Go 0007166, Establishment Of Cellular Localization, Establishment Of Localization, Organelle Organization And Biogenesis, Regulation Of Biological Quality, Reproductive Process, Signal Transduction, Soluble Fraction
MX1	1.96	0.10	Cell Development, Cytoplasm, Defense Response, Positive Regulation Of Biological Process, Positive Regulation Of Cellular Process, Positive Regulation Of Developmental Process, Programmed Cell Death, Regulation Of Developmental Process, Signal Transduction
DEFA1	-5.19	0.10	Behavior, Immune Response, Immune System Process, Locomotory Behavior, Response To Chemical Stimulus, Response To External Stimulus
ATF2	-2.9	0.10	Rna Polymerase Ii Transcription Factor Activity, Transcription Factor Binding
CCL8	-2.79	0.10	Behavior, Cell Cell Signaling, Establishment Of Cellular Localization, Establishment Of Localization, Locomotory Behavior, Multi Organism Process, Response To Chemical Stimulus, Response To Virus, Signal Transduction
MMP3	-2.78	0.10	Cellular Macromolecule Metabolic Process, Cellular Protein Metabolic Process, Endopeptidase Activity, Extracellular Region, Extracellular Region Part, Extracellular Space, Peptidase Activity, Protein Metabolic Process
PTGS1	-2.73	0.10	Biosynthetic Process, Cell Fraction, Cellular Biosynthetic Process, Cytoplasm, Membrane Fraction, Nucleus, Regulation Of Biological Quality
MEF2D	-2.49	0.10	Anatomical Structure Development, Multicellular Organismal Development, Muscle Development, System Development
CSF1	-2.69	0.10	Cell Proliferation Go 0008283, Hemopoiesis, Immune System Development, Immune System Process, Multicellular Organismal Development, Positive Regulation Of Cell Proliferation, System Development
CXCR1	-4.13	0.10	G alpha (i) signaling event, GPCR ligand biding, Neutrophil degranulation
RAPGEF2	-2.66	0.10	MAPK family signaling cascades
NOS2	-2.66	0.10	Hemostasis, Interleukin-4 and 13 signaling

Top 20 differentially expressed gene of pre-and post *C.nutans* treatment in inflammatory panel. (cont.) (Genes were ordered by significant differentially expressed gene)

	**Log_2 Fold Change**	**Adjusted P-value**	**Gene Set**
LTB	-2.63	0.10	Biosynthetic Process, Cell Signaling, Cellular Biosynthetic Process, Cytokine Metabolic Process, Cytokine Production, Positive Regulation Of Cytokine Biosynthetic Process, Positive Regulation Of Translation, Regulation Of Protein Metabolic Process, Regulation Of Translation, Signal Transduction
FOS	-4.41	0.10	Biopolymer Metabolic Process, Defense Response, Dna Metabolic Process, Inflammatory Response, Nucleobasenucleosidenucleotide And Nucleic Acid Metabolic Process, Nucleus, Rna Biosynthetic Process, Rna Metabolic Process, Transcription Dna Dependent
CEBPB	-3.23	0.10	Biopolymer Metabolic Process, Defense Response, Immune Response, Immune System Process, Inflammatory Response, Nucleobasenucleosidenucleotide And Nucleic Acid Metabolic Process, Nucleus, Response To External Stimulus, Response To Stress, Rna Biosynthetic Process, Rna Metabolic Process, Transcription, Transcription Dna Dependent
CCL16	-2.57	0.10	Behavior, Cell Cell Signaling, Chemokine Activity, Chemokine Receptor Binding, Cytokine Activity, G Protein Coupled Receptor Binding, Locomotory Behavior, Receptor Binding, Response To Chemical Stimulus, Response To External Stimulus
